# A scoping review of nursing interventions of improving resilience among university students during online learning

**DOI:** 10.1186/s12912-025-03581-0

**Published:** 2025-07-14

**Authors:** Rohman Hikmat, Iyus Yosep, Ai Mardhiyah

**Affiliations:** 1 Master Program of Nursing, Faculty of Nursing, Universitas Padjadjaran, Sumedang, Jawa Barat Indonesia; 2 Nursing Department, Faculty of Health Science, Universitas Aisyiyah Bandung, Bandung, Jawa Barat Indonesia; 3 Department of Mental Health Nursing, Faculty of Nursing, Universitas Padjadjaran, Sumedang, Jawa Barat Indonesia; 4 Department of Pediatric Nursing, Faculty of Nursing, Universitas Padjadjaran, Sumedang, Jawa Barat Indonesia; 5 Faculty of Nursing, Universitas Padjadjaran, Jl. Raya Ir. Soekarno KM. 21, Hegarmanah, Jatinangor, Sumedang, West Java 45363 Indonesia

**Keywords:** Online learning, Resilience, Students

## Abstract

Covid-19 pandemic has caused a change in learning methods from offline to online. These changes lead to mental health problems to students when online learning. To overcome this problem, resilience is needed so that individuals can adapt to changes in learning methods during the covid-19 pandemic. The purpose of this review is to explore nursing interventions for improving resilience among university students during online learning and get the research gap. This study uses the scoping review method. Data or literature were searched through CINAHL, PubMed, and CINAHL databases. The keywords used in literature searching are “resilience” AND “student OR undergraduate student” AND “online learning OR distance education”. The criteria for articles in this study are full text, randomized control trial or quasi experiment research design, English language, population and samples of students, and the publication period of the last during and after covid-19 (2019–2025). The authors identified 14 articles discussing nursing interventions to improve resilience in university students during online learning. Five main types of interventions were identified: (1) resilience-based intervention programs, (2) mindfulness and yoga-based interventions, (3) positive psychology and journaling-based interventions, (4) social skills and problem-solving training, and (5) simulation-based and curricular interventions. These nursing interventions have been shown to significantly increase resilience, helping students adapt to the shift from offline to online learning. Additionally, the research revealed several gaps, including a lack of consensus on the optimal duration of interventions, limited diversity in study populations (mainly focusing on university students), and insufficient exploration of multidisciplinary approaches combining different therapeutic methods. Resilience is an important aspect to increase students’ resilience in facing academic pressure. Activities can be carried out online or offline by considering the client’s condition holistically.

## Introduction

Changes in learning methods for students from offline to online cause psychological problems in students. The problems occurs because students encounter with the new methods of learning that need a proper adaptation. In addition, the rapid change in learning methods causes students makes the students do not have a sufficient time to adapt to the new method [1]. The unpreparadeness may cause psychological problems that later may have short-term and long-term impacts [2]. Previous research argues that the impact of online learning is that students become passive, less creative and less productive; students experience stress; and increase students’ language literacy skills [3]. The negative impact of online learning include lack understanding the material, being lazy, signal interference, being bored, increased tasks and using a lot of data. Moreover, online learning affects the psychological states such as anxiety, stress and depression [4]. 

The prevalence of mental health problems during online learning is also increasing. Previous research showed that there were 7,143 students who experienced severe, moderate and mild anxiety [5]. In addition to anxiety, 64.7% of respondents experienced psychological problems with the proportion of 64.8% experiencing anxiety, 61.5% experiencing depression, and 74.8% experiencing trauma [6]. Students in Spain showed that 72% of adolescents aged 18 years and over experienced psychological distress [7], 90.4% of children and adolescents aged 6–15 years in China experienced depression and anxiety [8]. Study in Indonesia showed that 59.5% of adolescents aged 15–18 years experienced psychological disorders [9]. The number of problems shows the need for efforts to reduce mental health problems in students.

Nurses have a role as school nurses to improve the quality of student health including improving mental health. Nurses have a role to support and improve student resilience [10]. Nurses act as health agents in identifying, managing, and responding to challenges to student wellbeing, especially in reducing stress, anxiety, and depression issues that can arise during the online learning process [11]. Nurses can provide counseling services, mental health education, and emotional support specifically tailored to the needs of students. In addition, they can facilitate psychosocial skills training programs, including stress management techniques, increased independence, and improved adaptability to changes in technology-based learning environments [12]. 

Furthermore, an unexplored dimension lies in understanding the intricate relationship between students’ technological competence and their resilience in online learning contexts. This research gap underscores the importance of this scoping review, as it presents an opportunity to synthesize existing knowledge, identify gaps, and provide the foundation for a more comprehensive understanding of how nursing interventions can effectively nurture student strengths and resilience in the dynamic landscape of online education.

Previous study has demonstrated that nursing intervention can help students minimize stress and despair [13]. Efforts to alleviate stress and depression are being made by building resilience. As a result, nurse treatments to improve resilience are required in order to reduce mental health problems in students. Previous research has shown that students need resilience to adjust to changes in lecture styles [10]. However, nurses’ roles in providing interventions to increase student resilience have not been optimal. As a result, this is the first study to examine nurse treatments to promote student resilience during online learning.

Previous scoping reviews on nursing interventions have been shown to reduce stress and depression in students during online learning [14]. Efforts to reduce stress and depression are by increasing student resilience. Therefore, a scoping review is needed to describe nursing interventions to increase resilience in students during online learning. Student resilience is key in enhancing student adaption to online learning. As a result, nurse treatments to build resilience are crucial in enhancing student resilience. So that students can overcome the psychological challenges that come with online learning. The purpose of this scoping review is to explore nursing interventions for improving resilience among students during online learning and get the research gap.

## Materials and methods

### Design

This scoping review utilized the methodological framework originally proposed by Arksey and O’Malley and subsequently refined by Levac et al., to ensure greater clarity and rigor in each stage of the review process [15, 16]. A scoping review is a methodological approach designed to map key concepts, types of evidence, and research gaps in a defined field through a systematic and transparent process [17]. As a broad conceptual framework, it is particularly useful for examining complex or heterogeneous literature related to the research topic [18].

Following the enhancements suggested by Levac et al., the review was conducted through six stages: (1) identifying the research question; (2) identifying relevant studies through a comprehensive search across databases and gray literature; (3) selecting studies based on clear inclusion and exclusion criteria; (4) charting the data systematically; (5) collating, summarizing, and reporting the results; and (6) incorporating consultation with experts to enhance the methodological robustness [16].

To guide the reporting and ensure transparency, we also applied the PRISMA-ScR (Preferred Reporting Items for Systematic reviews and Meta-Analyses extension for Scoping Reviews) checklist. The central research question for this review was: “How can nursing care delivery be utilized to enhance student resilience in the context of online learning, and what research gaps remain in this area?”

### Search methods

The authors used three databases to search for articles, namely PubMed, CINAHL, and Scopus. The major keywords used in this study are: resilience, nursing interventions, online learning, students. Searching strategy in this study used PRISMA for Scoping Review to select articles on nursing interventions to improve resilience in students during online learning. Searching strategy in this study used:

PubMed: ((“psychological resilience“[MeSH Terms] OR “resilience“[TW] OR “resiliences“[TW] OR “resiliency“[TW] OR “academic resilience“[TW]) AND (“nursing interventions“[TW] OR “nursing care“[MeSH Terms] OR “nursing care“[TW] OR “nursing care delivery“[TW])) AND ((“student“[MeSH Terms] OR “students“[TW] OR “undergraduate student“[TW] OR “university students“[TW] OR “college students“[TW])) AND ((“distance education“[MeSH Terms] OR “online learning“[TW] OR “online education“[TW])).

Scopus: TITLE-ABS-KEY (“psychological resilience” OR “resilience” OR “resiliences” OR “resiliency” OR “academic resilience”) AND TITLE-ABS-KEY (“nursing interventions” OR “nursing care” OR “nursing care delivery”)AND TITLE-ABS-KEY (“student” OR “students” OR “undergraduate student” OR “university students” OR “college students”)AND TITLE-ABS-KEY (“distance education” OR “online learning” OR “online education”).

CINAHL: TX (“psychological resilience” OR “resilience” OR “resiliences” OR “resiliency” OR “academic resilience”) AND TX (“nursing interventions” OR “nursing care” OR “nursing care delivery”) AND TX (“student” OR “students” OR “undergraduate student” OR “university students” OR “college students”) AND TX (“distance education” OR “online learning” OR “online education”).

### Inclusion and exclusion criteria

To search for articles, the author used PCC’s framework to get a concept for determining terms:

#### Population:

University students from various fields of study.

#### Concept:

Health-promoting interventions provided or initiated by nurses.

#### Context:

Improving resilience in the context of online learning.

In this review, nursing interventions are broadly defined as evidence-based actions or strategies that are initiated, delivered, or facilitated by nurses to promote psychological well-being and enhance resilience among university students. These may include educational programs, relaxation techniques, counseling, psychoeducation, or stress-reduction activities. While some interventions such as yoga or mindfulness may also be facilitated by other health professionals, studies were included if nurses were involved in the intervention process or if the intervention could be reasonably adopted into nursing practice as part of a holistic and promotive mental health approach.

Inclusion criteria were established to ensure the relevance, focus, and methodological rigor of the selected studies. This review included experimental studies, such as quasi-experimental designs and randomized controlled trials (RCTs), as these methodologies provide strong evidence regarding the effectiveness of interventions. The population targeted in the selected studies comprised university students from any academic discipline, considering that the issue of resilience in the context of online learning is broadly relevant and not limited to nursing students. The interventions included were health-promoting interventions delivered, initiated, or potentially adopted by nurses in clinical or educational settings. These interventions encompassed a range of strategies such as psychoeducation, stress management training, relaxation techniques, and mindfulness-based practices that align with the scope of nursing in promotive and preventive mental health care. Only studies published in English were considered, in line with the standard language of scientific publication. Furthermore, the publication period was limited to the last ten years (2019–2025) to ensure the inclusion of current and relevant literature. Studies were excluded if they were categorized as grey literature (e.g., dissertations, conference proceedings, or unpublished reports) or if only the abstract was available without access to the full text.

### Quality appraisal

Although quality appraisal is not a mandatory component of scoping reviews, it was included in this study to provide additional context regarding the methodological rigor of the included studies. The Joanna Briggs Institute (JBI) critical appraisal tools were used to assess the quality of the articles. Specifically, we applied the 13-item checklist for randomized controlled trials and the 9-item checklist for quasi-experimental studies. Each item on the checklist was rated with four possible responses: Yes (scored as 1), No, Unclear, and Not Applicable (all scored as 0).

A threshold of 75% was used as the minimum acceptable quality score for inclusion in the synthesis. Two authors (RH and IY) independently conducted the appraisal. Discrepancies between reviewers were resolved through discussion and consensus. If consensus could not be reached, a third author was consulted to provide an independent judgment.

### Data collection and analysis

**Fig. 1:**
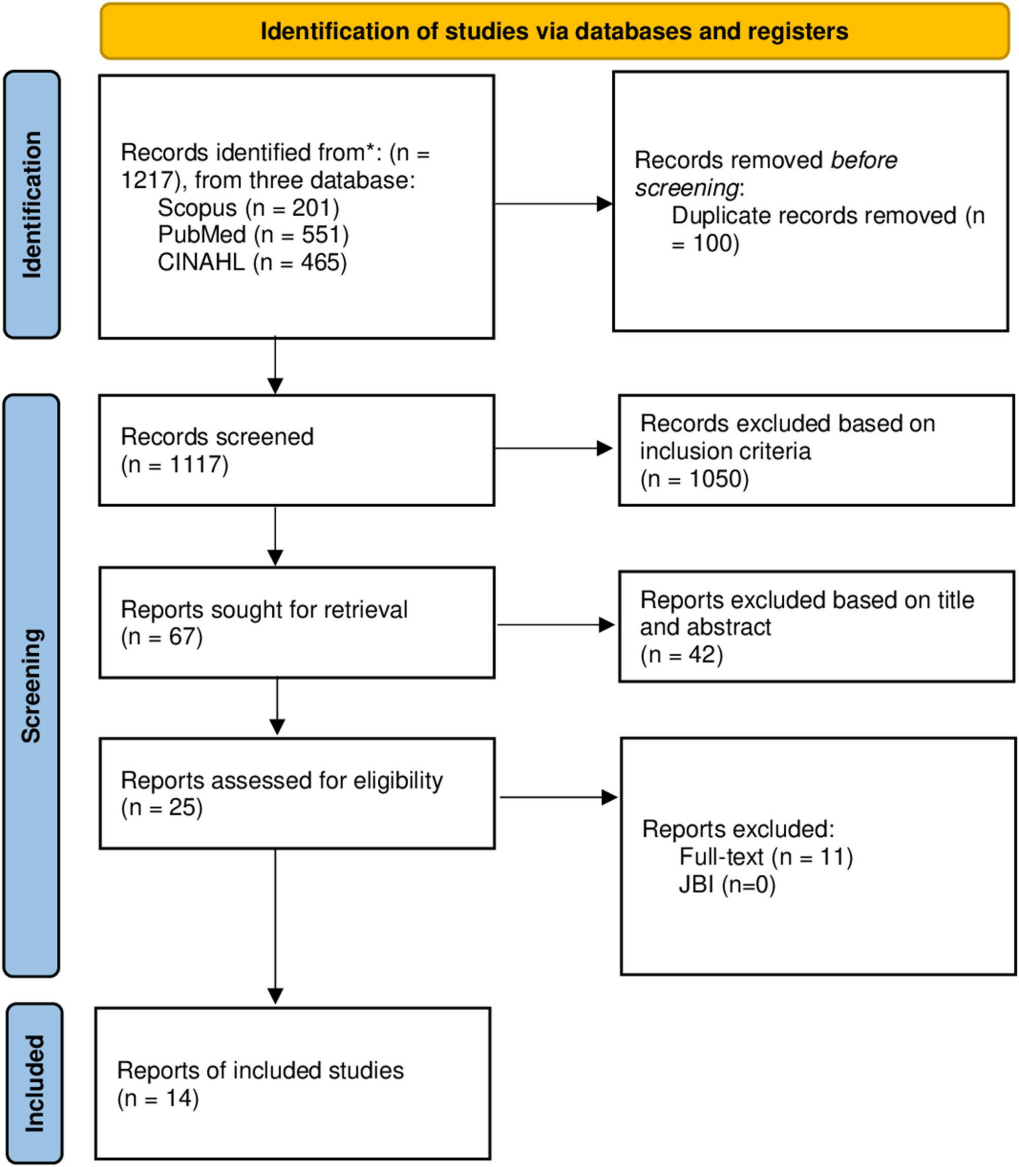
PRISMA flow diagram

The authors used PRISMA flowchart for selected publications for review by (1) detecting duplicate studies, (2) filtering titles and abstracts based on research aims, and (3) checking fulltext availability. Ekstraksi data menggunakan tabel manual berdasarkan hasil telaah penulis. The tabulation approach is performed manually by searching for research items such as author, country, study design, objectives, interventions, and results (Fig. 1). The qualitative descriptive approach was applied in this study’s article analysis. The authors discusses several studies concerning nursing treatments to enhance resilience. The author then categorizes the numerous types of nursing interventions discovered based on related interventions. Data extraction and data analysis were conducted by two independent and trained authors. Debates during the data extraction and analysis process were resolved by discussion and deliberation. Then, if there is still debate in the discussion related to the article, the third author is invited to provide an assessment of the article.

## Results

The systematic literature search initially identified 1,217 records across three databases: Scopus (*n* = 201), PubMed (*n* = 551), and CINAHL (*n* = 465). After removing 100 duplicate records, 1,117 unique articles underwent title/abstract screening, of which 1,050 were excluded based on inclusion criteria. The remaining 67 full-text articles were assessed for eligibility, resulting in 42 exclusions. Ultimately, 25 studies underwent full-text review, with 11 excluded after detailed evaluation. Fourteen studies met all inclusion criteria and were included in the final analysis (JBI-appraised studies: *n* = 0). The outcomes of the critical appraisal tools are presented in Table 1, demonstrating the articles’ quality, with those surpassing a 75% threshold deemed as meeting the criteria for inclusion in the review (Table 1).

**Table 1:** JBI critical appraisal tool

Authors, published year	JBI critical appraisal tool	Study design
(Akeman et al., 2020)	84,6% (11/13)	RCT
(DeTore et al., 2023)	84,6% (11/13)	RCT
(Galante et al., 2021)	84,6% (11/13)	RCT
(Enrique Roig et al., 2020)	76,9% (10/13)	RCT
(Bartos et al., 2020)	88,8% (8/9)	Quasi-experimental
(Mayor-Silva et al., 2021)	84,6% (11/13)	RCT
(Chang et al., 2022)	84,6% (11/13)	RCT
(Şenocak & Demirkıran, 2023)	76,9% (10/13)	RCT
(Lohner & Aprea, 2021)	100% (13/13)	RCT
(Litvin et al., 2023)	76,9% (10/13)	RCT
(Cabañero-Martínez et al., 2023)	88,8% (8/9)	Quasi-experimental
(Wadi et al., 2024)	88,8% (8/9)	Quasi-experimental
(Pehlivan, 2024)	100% (9/9)	Quasi-experimental
(Kadian et al., 2022)	76,9% (10/13)	RCT

Table 2 presents a synthesis of 14 studies evaluating resilience-building interventions among university students using randomized controlled trials (RCTs) and quasi-experimental designs. The analysis of the fourteen included studies reveals contributions from nine countries: the USA [19–21], Spain [22–24], the UK [25, 26], Turkey [27, 28], Ireland [29], Germany [30], India [31], and one study with unspecified Middle Eastern/Asian origin [32]. The interventions varied in format, including face-to-face sessions, online modules, mobile apps, mindfulness training, journaling, yoga, and problem-solving skills development. Sample sizes ranged from 67 to 1,165 participants, with mean ages spanning 18.81 to 26 years (extending to 51 years in nursing cohorts). The studies encompassed diverse disciplines, including nursing (4 studies), medicine, business, music, physical therapy, and general undergraduate programs. Most studies using validated resilience and mental health questionnaires such as the Connor-Davidson Resilience Scale (CD-RISC), the Brief Resilience Scale (BRS), and the Depression Anxiety Stress Scale-21 (DASS-21). Across most studies, the interventions showed significant improvements in resilience, mental well-being, and reductions in depression, anxiety, or stress. However, a few studies reported mixed or non-significant outcomes, suggesting variability in effectiveness based on context, duration, or delivery method. Overall, these findings demonstrate the feasibility and positive impact of structured, skill-based resilience interventions for young adult learners. These interventions were not limited to nursing-led programs, but spanned multiple disciplines and approaches, including psychological training, mindfulness practices, mobile health technologies, simulation, journaling, yoga, and structured resilience curricula.

**Table 2:** Data extraction

No	Authors & Years	Objective	Country	Method	Sample	Questionnaire	Intervention	Results
1.	(Akeman et al., 2020)	To evaluate the impact of a short, universal resilience program on first-year college students.	USA	RCT	252 students with mean age 18.81	the Connor-Davidson Resilience Scale (CD-RISC 10)	The resilience program consisted of four weekly 50-minute sessions, led by clinical psychology postdoctoral fellows or doctoral students, with small group facilitation by trained undergraduate volunteers and research assistants. The program was developed and supervised by licensed clinical psychologists.	Resilience programming improved student resilience at semester-end, with significant reductions in resilience, perceived stress and depression (*p* < 0.05).
2.	(DeTore et al., 2023)	Evaluating the effectiveness of a transdiagnostic resilience training program in at-risk young adults.	USA	RCT	107 students (RT = 54; control = 53)	Beck Depression Inventory (BDI), Peters Delusions Inventory (PDI), mindfulness, self-compassion, and mentalization scales	The 4-week “Resilience Training (RT)” program consists of 4 sessions (1.5 h each, small groups). Session 1: Introduction to the concepts of resilience and mindfulness, mindfulness practice. Session 2: Learning and practicing self-compassion. Session 3: Mentalization training (understanding other people’s thoughts). Session 4: Integration of the three skills into everyday life and application practice.	RT program is effective in reducing symptoms of depression, anxiety, and distress related to psychotic experiences (PEs); increasing mindfulness, self-compassion, and resilience. Effects persist for up to 12 months.
3.	(Galante et al., 2021)	Offering mindfulness courses to university students enhances their resilience to stress for up to a year.	UK	RCT	616 college students	Routine Evaluation Outcome Measure (CORE-OM), Mental well-being was assessed with the 14-item Warwick- Edinburgh Mental Wellbeing Scale (WEMWBS)	The eight weekly sessions (75–90 min) included mindfulness meditation, reflection, and interactive exercises. Students practice mindfulness at home for 8–25 min daily, with both formal and informal practices. Missed sessions were followed up by email, and students could speak privately with the teacher if needed.	Resilience increased significantly in students who participated in the MSS program, with positive effects lasting up to one year (*p* < 0.05).
4.	(Enrique Roig et al., 2020)	Explored the feasibility of a web-based resilience program using positive psychology, with human or automated support, for college students.	Ireland	RCT	81 college students with mean age 26 years	Connor-Davidson Resilience Scale and Pemberton Happiness Index	The “Space for Resilience” is a 7-module web-based program designed to enhance resilience and well-being. It incorporates cognitive-behavioral techniques like cognitive flexibility, optimism, and active coping, along with social support, lifestyle, and values ​​education. Each module includes videos, quizzes, psychoeducation, personal stories, interactive activities, mindfulness exercises, homework, and goal-setting.	After the CRAFT program, students showed improved welfare and resilience (*p* < 0.05).
5.	(Bartos et al., 2020)	examined the CRAFT program’s benefits, combining mindfulness, yoga, positive psychology, and emotional intelligence, on the health of student musicians during lockdown.	Spain	Quasi-experimental	93 musician students with age > 18 years	Connor-Davidson Resilience Scale and Pemberton Happiness Index	The CRAFT program combines physical activity and relaxation through a 15-minute hatha yoga protocol, focusing on five elements and four foundations. Initially, it emphasizes consciousness, relaxation, and attention, followed by a broader focus in later classes. Practices like yoga, mindfulness, and the FEM meditation help student musicians develop self-awareness and emotional regulation.	Significant differences in resilience scores were observed, with higher resilience in the control group compared to the intervention group (*p* = 0.06).
6.	(Mayor-Silva et al., 2021)	Assessed the effectiveness of online versus face-to-face programs in improving coping strategies for resilience, considering personality traits, mood, and academic stress.	Spain	RCT	245 students of the Nursing and Physical Therapy Degree with mean age 19.7	the Positive and Negative Affect Schedule (PANAS), the Academic Stressors Scale, the Coping Orientation to Problems Experienced (COPE), the Connor-Davidson Resilience Scale, and the NEO-FFI scale	The RG intervention, delivered in one session, covered thought-stopping, positive emotions, social support, problem-solving, and relaxation techniques through a brief masterclass, reflections, and practical exercises.	The intervention resulted in a significant reduction in stress and an increase in well-being and resilience in students (*p* < 0.05).
7.	(Chang et al., 2022)	Evaluated the impact of brief online Isha Upa Yoga modules on undergraduates’ mental health.	USA	RCT	679 university students with mean age21.3	Brief Resilience Scale	The Online Isha Upa Yoga program included 25-minute video sessions with guided practices, followed by weekly surveys for 12 weeks. Participants received audio and written instructions for daily practices.	Post-training, students in the experimental group showed higher resilience and self-efficacy, and lower perceived stress at both post-training and follow-up (*p* < 0.05).
8.	(Şenocak & Demirkıran, 2023)	Investigated the effects of problem-solving skills training on nursing students’ resilience, stress levels, and self-efficacy.	Turkey	RCT	149 s-year nursing students with mean age 19.97	Resilience Scale for Nurses, Social Problem Solving-Inventory-Short Form, Perceived Stress Scale, and General Self-Efficacy Scale	The experimental group received eight weekly sessions (55–150 min) covering problem-solving skills, cognitive reappraisal, emotion communication, and goal setting, using lectures, group work, and role play.	The Resilience Journal intervention led to increased resilience in students (*p* < 0.05).
9.	(Lohner & Aprea, 2021)	Explored the potential of journal interventions to increase resilience in students.	German	RCT	100 business school university students with mean age 23.74	Children’s Social Self-Efficacy in Peer Interaction Scale	The resilience training used two journaling techniques: the Attention Version, where participants recorded daily challenges, and the Mastery Version, where they reflected on challenges they successfully overcame. These exercises aim to enhance attention and mastery.	A 6-month intervention increased resilience and decreased depression in students, with lasting improvements in self-esteem at 3 months (*p* < 0.05).
10	(Litvin et al., 2023)	Assessing the impact of the eQuoo mobile application on resilience, anxiety, and depression in college students.	UK	RCT	1165 students	Rugged Resilience Measure (RRM), Generalized Anxiety Disorder-7 (GAD-7), Patient Health Questionnaire-8 (PHQ-8)	The mobile application “eQuoo” was played for 5 weeks. A gamified app based on CBT, positive psychology, and narrative storytelling. Each week users complete a “book” with narrative and challenges. Features include: emotional skills training, growth mindset exercises, decision making, conflict resolution, and interpersonal relationship development. Players take on the role of “Lodestar” who is tasked with fighting “The Quavering” through psychological skill development.	The eQuoo app significantly increased resilience and decreased anxiety and depression compared to active and passive control groups. Retention of use was 64.5%, the highest compared to other groups.
11	(Cabañero-Martínez et al., 2023)	to evaluate the effectiveness of a standardized patient simulation program and to analyze to what extent the students transferred the skills covered in the simulation to clinical practice	Spain	Quasi-experimental	166 final year nursing students (Age 20–51, Mean = 23.44)	Attitudes Towards Medical Communication Scale, self-efficacy, communication skills, resilience	8 simulation sessions (1h15m) with standardized patients and complex care scenarios. Actors and instructors trained before hand.	Significant improvement in communication, self-efficacy, and resilience maintained over 6 months.
12	(Wadi et al., 2024)	to evaluate the implementation of SAR framework on medical students’ resilience, anxiety, depression, burnout, and academic stress.	Not stated (Middle East/Asia)	Quasi-experimental	149 Year 4 medical students (Age not specified)	the Medical Student Resilience Scale (MeRS), Academic Resilience Scale from the Medical Student Stressor Questionnaire (ARS/MSSQ), Depression Anxiety Stress Scales (DASS-21), and Copenhagen Burnout Inventory (CBI).	5-hour online workshop for course coordinators, with ongoing support. Students receive indirect intervention via modified curriculum.	Increased resilience (MeRS), reduced depression, anxiety, and stress (DASS-21, MSSQ).
13	(Pehlivan, 2024)	to evaluate a stress management course’s impact on nursing students’ resilience and coping styles; and to investigate the relationship between coping styles and resilience	Turkey	Quasi-experimental	67 nursing students (Mean age = 21.16)	Connor-Davidson Resilience Scale (CD-RISC) and the Stress Coping Styles Scale.	14-week elective course (2 h/week) including stress theory, coping techniques, question and answer (Q&A), games, role-playing, homework.	Significant increase in optimistic coping. No change in resilience or other coping styles.
14	(Kadian et al., 2022)	Evaluating the feasibility of a brief resilience intervention in college students	India	RCT	220 students	Brief Resilient Coping Scale (BRCS), Perceived Stress Scale (PSS), Patient Health Questionnaire-4 (PHQ-4)	The brief resilience intervention consisted of 2 sessions (30 min each) at a 2-week interval. Session 1: Discussion on the meaning of resilience, creativity in overcoming difficult situations, the importance of vision and hope, active and passive coping strategies. Session 2: Exercise in setting life goals (SMART goals), assessing life priorities, strengthening protective factors such as social support, positive thinking, and self-talk. The control group only received an educational pamphlet on resilience.	No significant differences were found between the intervention and control groups. However, the intervention showed potential in improving coping and resilience.

Most interventions aimed to improve mental health outcomes such as stress, anxiety, depression, and self-efficacy. The majority of studies (12 out of 14) reported statistically significant improvements in resilience and related mental health indicators (*p* < 0.05), indicating the effectiveness of these interventions (Table 2). The interventions can be categorized into five main approachs:

### Resilience-based intervention programs

This theme includes structured programs specifically designed to enhance resilience among university students through psychoeducation, experiential exercises, and self-regulation techniques. For example, 6-week Resilience and Self-Compassion (RSC) program comprising weekly sessions that incorporated didactic teaching, experiential activities such as inner critic work and self-kindness practice, and group discussions [19]. The program was delivered by a licensed clinical psychologist. Similarly, 4-session Resilience Training (RT) using online platforms, which included core components such as mindfulness, mentalization, and self-compassion exercises aimed at managing emotional distress [20]. 7-week resilience program that targeted emotion regulation, personal growth, and psychological flexibility [31]. These programs were generally facilitated by psychologists, counselors, or trained mental health professionals. While nurses were not the primary facilitators, they can play a vital supportive role in implementation, particularly by identifying at-risk students, referring them to appropriate services, and integrating resilience content into nursing student wellness workshops.

### Mindfulness and yoga-based interventions

This theme includes interventions that utilize mindfulness meditation and yoga practices to strengthen present-moment awareness, emotion regulation, and stress reduction as key pathways to resilience. For example, 8-week Mindfulness Skills for Students (MSS) intervention that included 90-minute weekly sessions led by certified mindfulness instructors [25]. The program involved formal meditation, breathing techniques, and reflection exercises. 4-week yoga-based intervention called Isha Upa Yoga, which was delivered through daily 25-minute video-guided sessions focusing on posture, breath control, and energy regulation [21]. 5-week mindfulness program with weekly online sessions incorporating mindful breathing, body scans, and guided imagery [22]. The facilitators in these interventions were trained yoga instructors or mindfulness practitioners. Nurses may adopt a complementary role, especially in university health centers or student wellness units, by incorporating mindfulness techniques into stress management programs or organizing workshops alongside certified instructors. Nurse educators may also integrate these practices into nursing curricula to improve student coping mechanisms.

### Positive psychology and journaling-based interventions

Interventions under this theme aim to enhance students’ resilience through reflective writing, gratitude practices, and strengths-based cognitive reframing. Online program called *Space for Resilience*, which provided self-guided modules over 6 weeks including gratitude lists, future self-visualization, and values-based goal setting [29]. The students ask to write daily journals for 4 weeks, encouraging them to reflect on personal challenges, their emotional reactions, and effective responses [30]. Likewise, 5-week reflective journaling intervention that helped participants build insight into their coping mechanisms and self-growth [24]. These interventions were typically designed and supervised by psychology researchers or faculty. In these contexts, nurses could serve as facilitators or mentors, particularly within nursing education, by guiding students in structured reflection or resilience journaling as part of professional development or self-care assignments.

### Social skills and problem-solving training

This theme highlights interventions that aim to enhance emotional communication, assertiveness, and interpersonal problem-solving abilities, which are foundational to psychological resilience. Six-session group training that focused on emotional expression, active listening, and peer communication through interactive activities and role-plays [27]. 8-session psychoeducational program that taught cognitive restructuring, problem-solving strategies, and coping skills through a structured curriculum [28]. These programs were led by mental health nurses and clinical psychologists, emphasizing the critical role of nurses not only as facilitators but also as educators and mental health promoters. Nurses in this theme directly led the sessions, evaluated participants’ progress, and provided emotional support, thereby reinforcing their role as active agents in resilience-building.

### Simulation-based and curricular interventions

The final theme encompasses educational innovations embedded into the formal curriculum or simulation-based clinical learning, aiming to foster resilience indirectly by enhancing coping skills, self-efficacy, and reflective thinking. Clinical simulation program with debriefing over 3 sessions, where students encountered high-stress scenarios in a safe environment and reflected on their reactions [24]. Structured Academic Resilience (SAR) curriculum integrated into one semester of nursing education, covering topics such as resilience theory, peer support, and reflection exercises [32]. These interventions were delivered by nurse educators and simulation instructors. The role of nurses was central, as they designed the curriculum, facilitated simulation experiences, and guided students through reflective debriefings. By integrating resilience into core nursing education, these programs reinforce students’ adaptive capacities in both academic and clinical settings.

### Research gap

Although interventions to improve resilience have shown significant results, several research gaps remain. One key issue is the lack of consensus on the optimal duration and intensity of interventions; while some studies report success with short-term programs, others utilize longer formats, making it unclear which approach ensures maximum effectiveness. Interventions that are too brief may lack impact, while extended ones risk reduced participant adherence. Furthermore, most studies have focused on relatively homogeneous populations, primarily university students, limiting the generalizability of findings to broader demographic groups such as high school students, workers, or the general public. Another underexplored area is the application of multidisciplinary approaches. While some evidence supports the effectiveness of combining mindfulness, yoga, and positive psychology, research on structured collaboration among professionals from diverse disciplines such as nurses, psychologists, and yoga instructors is still limited. Such integration may offer more holistic and sustainable resilience-building interventions, especially when tailored to culturally and contextually diverse populations.

## Discussion

Students have mental health problems occurs as a result of the move from offline to online schooling during the COVID-19 outbreak. The most common mental health concerns are stress, anxiety, and depression. The resiliency of the students resilience may help them enhance their mental health and acclimatize to online learning. Resilience is an individual’s response to emotional, cognitive, and behavioral challenges [33]. Increasing student resilience has become an important focus in supporting them to cope with challenges, particularly those arising from online learning and academic pressure. These situations often cause significant stress, affecting their mental wellbeing and ability to adapt. A range of interventions have been developed to help students build resilience, including programs based on positive psychology, mindfulness, yoga, and problem-solving skills development. These interventions are designed to provide effective coping strategies, improve emotional regulation, and promote adaptive capacity in the face of stressful situations [14, 34]. These findings underscore the importance of structured, evidence-based approaches to helping students cope with the complex academic pressures and challenges of modern education [35].

The role of nurses in enhancing resilience among university students has gained increasing recognition, particularly within the context of student mental health promotion and academic retention. Evidence from previous studies demonstrates that nurses contribute significantly not only as facilitators of structured resilience training programs but also as mentors, educators, and advocates within the academic environment. In interventions such as psychoeducation and problem-solving training [36], mental health nurses have led sessions directly, using therapeutic communication skills and evidence-based techniques to foster emotional regulation and adaptive coping. In simulation-based and curriculum-integrated programs [37, 38], nurse educators have been instrumental in embedding resilience-building content into clinical learning, thereby equipping students with practical tools to navigate academic and professional stressors. The urgency of this role is underscored by the high prevalence of anxiety, burnout, and psychological distress among university students, especially those in nursing and health-related fields who are frequently exposed to emotionally demanding learning environments [39]. As accessible, trusted health professionals within academic institutions, nurses are uniquely positioned to integrate resilience support into both formal education and informal student interactions, making them essential agents in the development of psychologically resilient future professionals [40].

Resilience-based training programs are structured interventions that aim to strengthen students’ coping mechanisms through psychoeducation, self-reflection, and emotional regulation strategies [19]. Mindfulness and yoga interventions focus on cultivating present-moment awareness, reducing stress, and enhancing psychological flexibility through meditation, breathing techniques, and physical postures [22]. Internet-based interventions, including online learning modules and mobile health platforms, offer flexible and scalable delivery of resilience content, making them especially relevant in remote or online learning settings [27, 41]. Students who regularly engage in mindfulness practices and possess secure attachment tendencies are better equipped to regulate emotions, stay focused during adversity, and apply adaptive coping strategies.

Resilience-based training programs are structured interventions that aim to improve individuals’ ability to adapt positively in the face of adversity through psychoeducation, emotional regulation techniques, and experiential activities. These programs typically involve sessions that combine cognitive-behavioral components, stress management skills, and reflective exercises to promote self-awareness and psychological flexibility [42]. For instance, a six-week Resilience and Self-Compassion (RSC) program demonstrated significant improvements in emotional regulation and stress reduction among students [43]. Delivered by mental health professionals, such programs emphasize internal coping resources, which are particularly vital for university students facing academic pressure and transitions. While nurses may not always serve as facilitators, they are well-positioned to identify at-risk students and refer them to such resilience-building initiatives, especially in campus health or wellness settings [44].

Mindfulness and yoga-based interventions target psychological resilience by enhancing present-moment awareness, self-regulation, and stress management. These programs often involve mindfulness meditation, breathing techniques, and yoga postures, which are practiced regularly to foster emotional balance and reduce physiological symptoms of stress. An 8-week Mindfulness Skills for Students (MSS) program and a 4-week yoga intervention both showed positive effects on anxiety and emotional well-being. Research has consistently linked mindfulness practices with reduced rumination, improved attention, and greater psychological flexibility [44, 45]. Such interventions are particularly useful in the context of online learning or during periods of isolation, such as the COVID-19 pandemic [46]. In nursing education, mindfulness practices can be embedded into curricula to promote student self-care and support clinical performance under pressure [27].

Interventions rooted in positive psychology, including reflective journaling and gratitude practices, aim to foster optimism, self-efficacy, and meaning-making core components of resilience. Programs like the *Space for Resilience* encouraged students to engage in daily reflection, write gratitude lists, and set values-based goals. Similarly, reflective journaling has been shown to enhance emotional processing, insight, and resilience in times of stress [41]. These low-cost, accessible strategies promote self-regulation and emotional expression, which are particularly important for students lacking formal mental health support [26]. In nursing and health education, journaling can be employed as a reflective tool within clinical placements, helping students process complex emotional experiences and enhance professional development [28]. Facilitators, including nurse educators, can guide students in structured reflection that supports both academic growth and personal well-being [47].

Social and interpersonal problem-solving interventions focus on developing emotional communication, assertiveness, and adaptive coping strategies through structured group sessions and role-plays. Such skills are fundamental to resilience, particularly in collaborative learning and clinical environments. A six-session emotional communication workshop and an eight-session cognitive restructuring and coping curriculum both reported improvements in students’ ability to navigate interpersonal stressors [48]. These interventions are often led by nurses or psychologists and have demonstrated success in enhancing students’ emotional intelligence and decision-making skills. Within nursing education, such training is critical for preparing students to manage real-world challenges, including patient interactions, teamwork, and ethical dilemmas [30]. By actively participating in such interventions, students gain greater confidence and competence in both academic and clinical settings [49].

The use of technology in delivering resilience content through online modules, mobile apps, or integrated curricula offers scalable, accessible, and context-sensitive methods for supporting student well-being. Internet-based interventions, such as asynchronous resilience modules or mobile-based mindfulness practices, have demonstrated effectiveness in reducing stress and promoting self-directed coping strategies [50]. These tools are particularly useful for reaching students in remote areas or those unable to attend in-person sessions. Furthermore, embedding resilience education into academic curricula as seen in simulation-based programs and structured academic resilience (SAR) curricula ensures that all students receive foundational training in adaptive functioning [34]. Nurse educators and curriculum designers play a crucial role in these approaches by integrating resilience-focused content into coursework, promoting reflective debriefings, and creating psychologically safe learning environments that mirror real-life healthcare challenges [51].

Individual resilience can be improved by using the internet. The advancement of technology provides an opportunity for health professionals to create online health services. Users can obtain varied content through online interventions at any time. Previous study has shown that online intervention is an effective strategy for reducing mental health problems in college students while they are learning online [52]. Another study found that digital-based nursing interventions can help pupils with anxiety and sadness during the Covid-19 epidemic [19, 53]. As a result, online interventions have the potential to deliver online nursing interventions for resilience building [54]. While the Covid-19 epidemic, students with strong resilience can remain calm in the face of problems and barriers while online learning. Positive activities such as doing entertainment activities at home, conversing with family members, eating together, or discussing ideas can boost resilience [55, 56]. Environmental variables and individual qualities can both influence resilience [51, 57, 58]. This suggests that the environment has an impact on student resilience.

Universities have a critical role in fostering resilience among students, particularly during their transition into higher education, which is often marked by increased stress and adjustment challenges. Integrating resilience training into student orientation or onboarding programs has been shown to be effective in promoting early adaptation and psychological preparedness [19]. Resilience training significantly improved students’ coping abilities and reduced perceived stress, particularly when introduced at the start of the academic journey. In addition, regular mental health workshops that include mindfulness, stress management, and problem-solving skills have been shown to not only reduce symptoms of anxiety and depression [34]. The incorporation of digital platforms for emotional check-ins and reflective journaling aligns with current trends in technology-assisted interventions, which have proven effective in increasing engagement and mental health self-monitoring [44]. Collectively, these strategies underscore the importance of a comprehensive, institutional approach to building student resilience, supported by evidence from multiple domains of research.

Policymakers in higher education and health sectors hold a strategic position in shaping the structural and policy-level frameworks needed to promote student resilience. The development of national guidelines for mental health and resilience education is essential to ensure consistent standards across institutions [46]. Policy-driven mental health promotion in universities can significantly reduce the long-term burden of mental disorders and improve academic retention [59]. Moreover, interdisciplinary mental health initiatives have demonstrated effectiveness in delivering holistic, innovative, and scalable interventions. Digital mental health programs with interprofessional collaboration significantly improved access and outcomes in university settings [60]. Policymakers should also prioritize funding and capacity-building for such programs to ensure sustainability and reach.

Nursing educators play a pivotal role in fostering resilience among nursing students, particularly given the emotionally demanding nature of clinical education and practice. Embedding resilience-building modules within the nursing curriculum has been shown to enhance students’ psychological preparedness and reduce the risk of burnout. Structured resilience education in nursing programs significantly improved students’ self-efficacy and capacity to manage clinical stress [61]. Additionally, training nurse educators and mentors to recognize signs of low resilience is essential, as early identification allows for timely interventions and referrals to appropriate support systems. Supportive educator-student relationships and early detection of mental health concerns are key factors in promoting nursing student retention and well-being [62]. Furthermore, facilitating interprofessional collaboration by involving nursing students in resilience-building activities alongside peers from other health disciplines encourages the development of empathy, shared coping strategies, and team-based problem-solving skills [27]. Collectively, these strategies position nurse educators not only as instructors, but also as mental health promoters and advocates within academic settings.

### Limitations

This study has several limitations in assessing student resilience during online learning in the Covid-19 pandemic. The limited nature of online learning makes it difficult to objectively measure resilience, as experiences vary and are influenced by home environments and stress levels. The small number of studies and lack of focus on specific factors related to online learning further restrict generalization. Most research also involves homogeneous student populations, reducing applicability to diverse groups. Inconsistent duration and intensity of interventions add uncertainty about what works best. Future studies should explore multidisciplinary approaches to provide more holistic support for resilience development.

## Conclusion

Based on the findings in this review, there are 14 articles that examine nursing interventions to improve student resilience during online learning. All articles indicate that nursing interventions can improve student resilience in facing online learning. Resilience is needed to improve students’ ability to adapt to changes in learning methods that are carried out online. Nursing intervention activities in this study are grouped into four types, namely resilience-based training, mindfulness, yoga, and problem-solving skills development. Nurses provide comprehensive nursing care from assessment to evaluation by expanding knowledge, building resilience, problem-solving skills, and providing emotional support to students who face challenges in online learning.

The implication of this study is that there is evidence that can be used as a basis for universities, health professionals, and other stakeholders to collaborate in supporting students’ mental health during online learning. This collaboration can involve the implementation of various nursing interventions, such as resilience-based training, mindfulness, yoga, and problem-solving skills development, which have been shown to be effective in improving student resilience. In addition, parents can also play an active role as companions or monitors in the process of online learning for students, as well as helping to create an environment that supports their emotional well-being. For further development, a systematic review and meta-analysis approach can be used to more comprehensively evaluate the impact of nursing interventions in improving student resilience and mental health in the context of online learning.

## Data Availability

All data generated or analysed during this study are included in this published article.
